# The Unexplored Role of Mitochondria-Related Oxidative Stress in Diverticular Disease

**DOI:** 10.3390/ijms25179680

**Published:** 2024-09-06

**Authors:** Martina Cappelletti, Lucia Pallotta, Rosa Vona, Antonella Tinari, Annalinda Pisano, Giovanni Casella, Daniele Crocetti, Dominga Carlomagno, Ivan Tattoli, Carla Giordano, Paola Matarrese, Carola Severi

**Affiliations:** 1 Department of Translational and Precision Medicine, Sapienza University of Rome, Viale del Policlinico, 155, 00161 Rome, Italy; m.cappelletti@uniroma1.it (M.C.); lucia.pallotta@uniroma1.it (L.P.); dominga.carlomagno@uniroma1.it (D.C.); ivan.tattoli70@gmail.com (I.T.); 2 Center for Gender-Specific Medicine, Italian National Institute of Health, Viale Regina Elena 299, 00161 Rome, Italy; rosa.vona@iss.it (R.V.); antonella.tinari@iss.it (A.T.); paola.matarrese@iss.it (P.M.); 3 Department of Radiological, Oncological and Pathological Sciences, Sapienza University of Rome, Viale del Policlinico, 155, 00161 Rome, Italy; annalinda.pisano@uniroma1.it (A.P.); carla.giordano@uniroma1.it (C.G.); 4 Department of Surgical Science, Sapienza University of Rome, Viale del Policlinico, 155, 00161 Rome, Italy; giovanni.casella@uniroma1.it (G.C.); daniele.crocetti@uniroma1.it (D.C.)

**Keywords:** oxidative imbalance, mitochondria, smooth muscle, antioxidants

## Abstract

The pathophysiology of diverticular disease (DD) is not well outlined. Recent studies performed on the DD human ex vivo model have shown the presence of a predominant transmural oxidative imbalance whose origin remains unknown. Considering the central role of mitochondria in oxidative stress, the present study evaluates their involvement in the alterations of DD clinical phenotypes. Colonic surgical samples of patients with asymptomatic diverticulosis, complicated DD, and controls were analyzed. Electron microscopy, protein expression, and cytofluorimetric analyses were performed to assess the contribution of mitochondrial oxidative stress. Functional muscle activity was tested on cells in response to contractile and relaxant agents. To assess the possibility of reverting oxidative damages, N-acetylcysteine was tested on an in vitro model. Compared with the controls, DD tissues showed a marketed increase in mitochondrial number and fusion accompanied by the altered mitochondrial electron transport chain complexes. In SMCs, the mitochondrial mass increase was accompanied by altered mitochondrial metabolic activity supported by a membrane potential decrease. Ulteriorly, a decrease in antioxidant content and altered contraction–relaxation dynamics reverted by N-acetylcysteine were observed. Therefore, the oxidative stress-driven alterations resulted in mitochondrial impairment. The beneficial effects of antioxidant treatments open new possibilities for tailored therapeutic strategies that have not been tested for this disease.

## 1. Introduction

Diverticular disease (DD) is a colonic pathology, with its incidence rapidly increasing and whose clinical presentation ranges from the most frequent asymptomatic diverticulosis (80%) to symptomatic uncomplicated (15%) and complicated (5%) DD [1]. Its controversial multifactorial etiopathogenesis, affecting the scarce efficacy of the actual therapeutic strategies, still remains to be elucidated [1,2,3]. Cardinal events are an increase in intraluminal pressure that promotes the formation of pseudo-diverticula that pour out from the colonic wall [4]. Through a systematic and detailed morphological and molecular analysis performed in parallel on patients with asymptomatic diverticulosis and complicated DD, it has been recently demonstrated in colonic smooth muscle specimens, in both clinical DD phenotypes, cell hyperplasia and disarray, paralleled by interstitial fibrosis, mainly affecting longitudinal muscle, that likely account for the reduced compliance of the colonic wall, highlighted by a decrease in maximal contraction and relaxation. The associated predominant biochemical feature was a tissue oxidative imbalance with a loss in antioxidant defenses, not strictly related to inflammation [5]. The trigger of the observed oxidative imbalance was hypothesized to be ischemia/reperfusion injury rather than inflammation. Transient compressions of vasa recta in the neck of the diverticulum might be easily promoted by the increased motility indices and high-amplitude propagated contractions, which characterize colons affected by all clinical DD phenotypes [6,7], the thicker muscle layers requiring increased oxygen consumption ending in further ROS production. These events could potentially influence the blood efflux in the diverticula region, which leads to recurrent chronic ischemia and, consequently, mitochondrial-related oxidative damage. In any case, the exact mechanisms underlying the oxidative imbalance and relatively ineffective antioxidant response still need to be elucidated. The link between oxidative stress and mitochondria is a crucial topic in cellular biology and pathology. Mitochondria, being the primary producers of ROS during energy production, are particularly susceptible to oxidative damage, which can impair their function and contribute to various diseases [8]. Managing oxidative stress through antioxidants, lifestyle changes, and potentially pharmacological agents is a promising approach to preventing or mitigating disease progression [9,10].

Considering the central role of mitochondria in cell metabolism and oxygen radical production, the objective of this study is to elucidate the dysfunctional mitochondrial pathways contributing to the progression of distinct DD clinical phenotypes. Achieving this aspect will improve our knowledge of disease pathogenesis and help identify new potential therapeutic targets. Moreover, keeping this in mind, the beneficial effects of antioxidant N-acetylcysteine in reverting oxidative-related cellular alterations were evaluated.

## 2. Results

### 2.1. Human Tissue Collection

Clinical information about human tissues collected from human sigmoid colons are shown in Table 1.

### 2.2. Ultrastructural Analyses of Mitochondria Smooth Muscle Tissues

**Figure 1:**
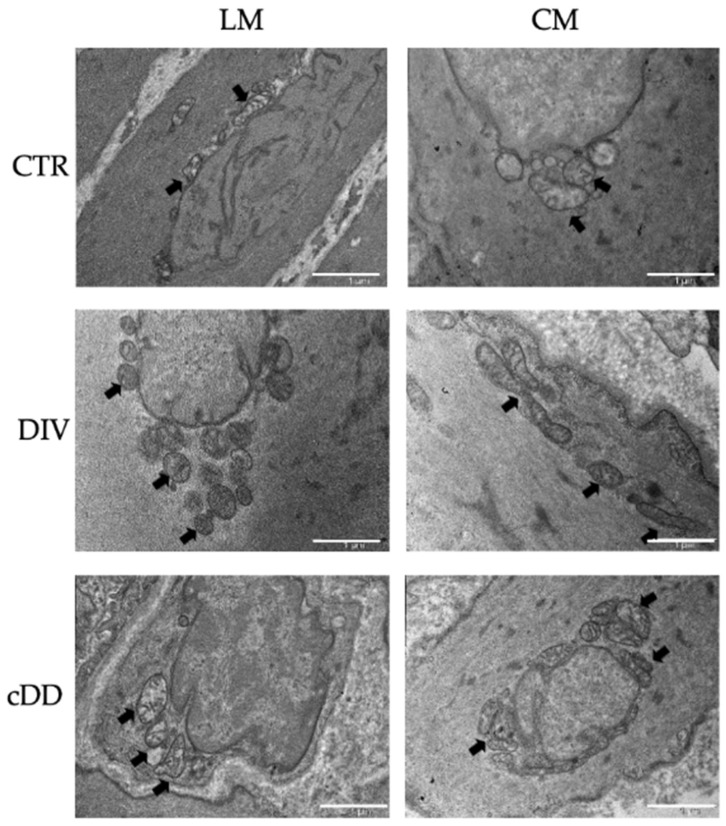
Transmission Electron Microscopy (TEM) analyses (bar = 1 µm). Ultrastructural analyses in CTR (control), DIV (diverticulosis), and cDD (complicated diverticular disease) of LM (longitudinal muscle) and CM (circular muscle) layers. Arrows indicate points of mitochondrial membrane fusion and elongated mitochondria.

Mitochondrial morphological analyses were performed by Transmission Electron Microscopy (TEM) to highlight possible ultrastructural alterations occurring in DD samples (Figure 1). As shown in Figure 1, both in the control and DD samples, mitochondria were distributed near the nucleus, indicating an active cellular metabolism [11]. In DD samples, independently from the clinical phenotypes, an abundant increase in mitochondria number was observed both in longitudinal and circular muscle layers, with visible points of mitochondrial membrane fusion (indicated by arrows) and elongated mitochondria possibly due to the dynamic process of adaptation to stressful conditions related to the pathology.

### 2.3. Mitochondrial Respiratory Chain

Mitochondria act as power stations of cells, taking advantage of energy derived from the mitochondrial electron transport chain (ETC) to catalyze the reaction of the reduction of ADP into ATP, which represents the cell’s energy currency. ETC includes five complexes, I, II, III and IV, and complex V, also called ATP synthase, each of which is constituted by the aggregation of more proteins. To evaluate the mitochondrial metabolic activity and biogenesis in response to cellular stress associated with diverticular disease, subunit A of complex II (SDHA) and subunit I of complex IV (COX) were evaluated. In detail, complex II is the only respiratory chain complex whose subunits are entirely encoded by nuclear DNA, whose number of copies remains stable for the entire cell’s life. On the contrary, the COX subunit is entirely encoded by mitochondrial DNA, whose number of copies is mutable in response to adaptation phenomena and thinly linked to mitochondrial number; consequently, it represents a good marker for mitochondrial biogenesis [12].

#### 2.3.1. Mitochondrial Respiratory Chain-Complex II (SDHA)

**Figure 2:**
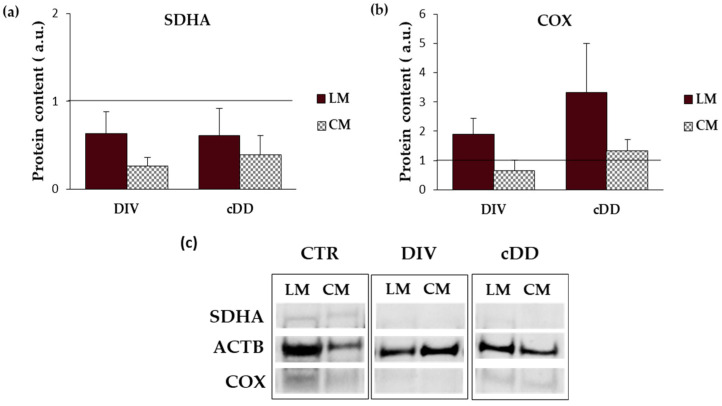
Mitochondrial respiratory chain complex investigation. (**a**) Mitochondrial respiratory chain—complex II (SDHA) (*n* = 4–8). (**b**) Mitochondrial respiratory chain—complex IV (COX) (*n* = 5–8). Data are expressed as mean ± SEM. β-actin (ACTB) was used as the internal control for normalization. Both analyses were performed in tissues by Western blot. (**c**) Representative Western blot. CTR: control, DIV: diverticulosis, cDD: complicated diverticular disease, CM: circular muscle, LM: longitudinal muscle.

In the controls, the content of SDHA was higher in the circular than longitudinal smooth muscle layer (LM: 0.43 ± 0.10; CM: 0.75 ± 0.21), whereas in DD samples, this difference between the two muscle layers was no longer evident. In fact, independently from the DD phenotype, the content of SDHA was decreased in comparison to the controls (Figure 2a,c and Supplementary Figure S1).

#### 2.3.2. Mitochondrial Respiratory Chain—Complex IV (COX)

Like SDHA, COX control content was also higher in the circular smooth muscle layer than in the longitudinal one (LM: 0.05 ± 0.03; CM: 0.15 ± 0.06). On the contrary, compared to the control, a progressive increase in COX was observed in the longitudinal muscle of DIV and cDD, while in the circular muscle layer, an increase in COX was appreciable only in cDD (Figure 2b,c and Supplementary Figure S1).

### 2.4. Mitochondrial Alterations and Antioxidant Status Evaluation in Isolated Smooth Muscle Cells

#### 2.4.1. Mitochondrial Mass

**Figure 3:**
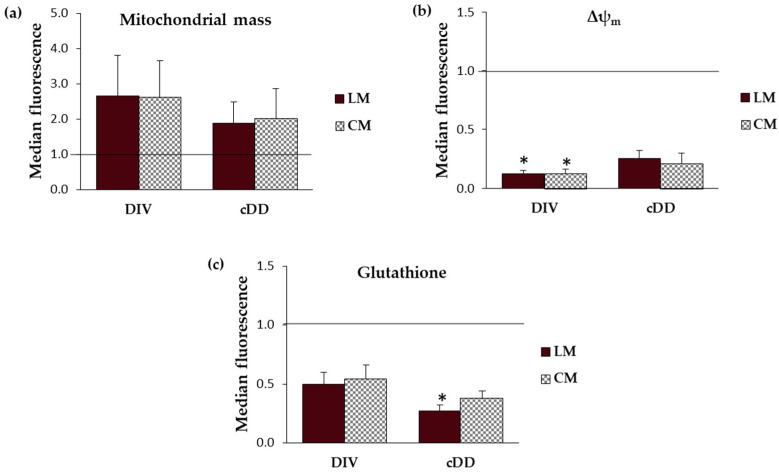
Cellular mitochondrial and oxidative parameter analyses. (**a**) Mitochondrial mass evaluation (*n* = 3–6). (**b**) Mitochondrial membrane potential (*n* = 4–6). (**c**) Total glutathione content evaluation (*n* = 8–10). Data are expressed as mean ± SEM. All analyses were performed in smooth muscle cells by cytofluorimetric analyses (* *p* ≤ 0.05 vs. control). CTR: control, DIV: diverticulosis, cDD: complicated diverticular disease, CM: circular muscle, LM: longitudinal muscle, ΔΨ_m_: mitochondrial membrane potential.

Mitochondrial biogenesis denotes the production of new mitochondria from pre-existing mitochondria, resulting also in an increase in mitochondrial mass, and can be considered a form of cellular adaptation in response to different stimuli, including oxidative insults [13]. Interestingly, differently from what was observed for the proteins of the respiratory chain, no quantitative differences were observed between the longitudinal and circular layers, regardless of the pathological condition. However, either in DIV or in cDD phenotypes, an increase in mitochondrial mass was observed in both muscle layers (Figure 3a).

#### 2.4.2. Mitochondrial Membrane Potential (Δψ_m_)

If cells can exploit the number of mitochondria to modulate their energy demand, the mitochondrial membrane potential can be monitored to evaluate the mitochondrial function. Regarding the controls, mitochondrial membrane potential evaluation did not highlight any significant difference between cells isolated from the circular muscle layer and those isolated from the longitudinal layer. On the contrary, in both pathological phenotypes, DIV and cDD, a significant decrease in mitochondrial membrane potential was observed in the cells of both circular and longitudinal muscle layers, compared to the respective controls (Figure 3b).

#### 2.4.3. Glutathione Intracellular Content

At last, the presence of oxidative stress can be indirectly revealed as the consumption of antioxidant defenses, such as glutathione, which represents the most abundant antioxidant molecule in cells [14]. We found that, in the controls, glutathione content was comparable between cells from longitudinal and circular muscles. A progressive and significant decrease in glutathione content was observed from the controls to the most advanced stages of the disease, i.e., from diverticulosis to cDD. This pattern was observed in cells isolated from both muscle layers (Figure 3c).

### 2.5. Functional Activity of Isolated Smooth Muscle Cells

To assess the cell’s functionality, the response to contractile and relaxant agents was evaluated in smooth muscle cells (Table 2). Regarding cell length, no differences were observed between the control and pathological samples either in the longitudinal or circular layer (Supplementary Materials Table S1). Instead, the resulting functional activity was impaired in the longitudinal muscle, and relaxation was reduced both in DIV and cDD, whereas contraction was reduced only in cDD.

### 2.6. Functional Effects of N-Acetylcysteine

**Figure 4:**
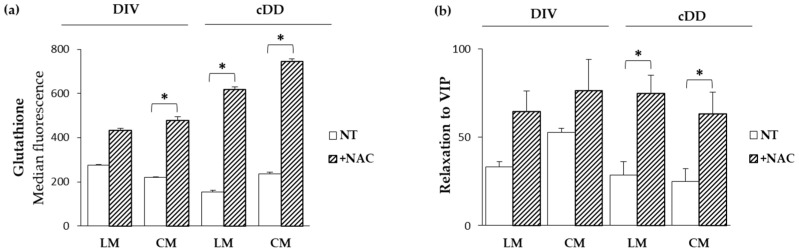
Functional effects of N-acetylcysteine. Samples were evaluated before and after 2 h of NAC incubation and compared with untreated samples. (**a**) Modulation of glutathione content in smooth muscle cells evaluated by cytofluorimetric analyses (*n* = 4–5). (**b**) Modulation of cellular functional activity in smooth muscle cells evaluated by micrometric assay (*n* = 2–4). Data are expressed as mean ± SEM. * *p* ≤ 0.05 vs. untreated samples. CTR: control, DIV: diverticulosis, cDD: complicated diverticular disease, NT: untreated samples, NAC: N-acetylcysteine.

N-acetylcysteine (NAC) is directly involved in the metabolism of glutathione, providing the cysteine necessary for its synthesis [15]. To investigate the potential beneficial role of NAC in reversing the oxidative damage and functional alterations observed both in diverticulosis and cDD, smooth muscle cells were incubated with NAC for two hours and the results were compared to samples of untreated cells. As expected, NAC was able to significantly increase glutathione content in cells from the circular layer of diverticulosis and both longitudinal and circular cells of cDD, compared to untreated cells (Figure 4a).

Despite no differences in contraction between the treated and untreated samples, NAC was able to reverse the decreased relaxant response and recover the functionality of treated cells. In detail, an increase in relaxant response was observed significantly after two hours of treatment in both longitudinal and circular layers of cDD (Figure 4b).

## 3. Discussion

**Figure 5:**
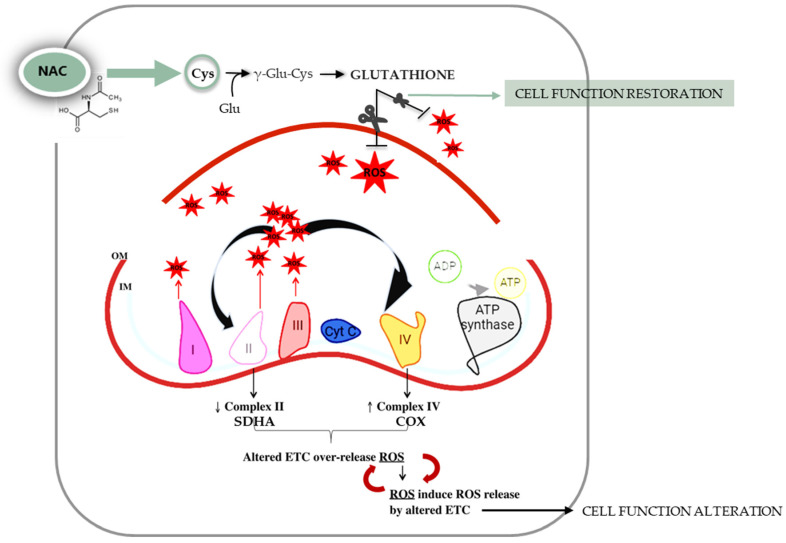
Mitochondrial electron transport chain (ETC). The ETC consists of protein complexes able to transfer electrons coming from oxidizable substrates from donors to acceptors with the final goal of producing adenosine triphosphate. In physiologic conditions during aerobic respiration, oxygen acts as the final electron acceptor and each complex of ETC includes specific sites for reactive oxygen species (ROS) release. Moreover, in pathological conditions, the overproduction of ROS negatively influence the ETC activity that in turn favors an ulterior ROS release. N-acetylcysteine is an antioxidant compound able to increase glutathione content by providing cysteine, a key precursor for its synthesis. IM: mitochondrial inner membrane, OM: mitochondrial outer membrane, ADP: adenosine diphosphate, ATP: adenosine triphosphate, NAC: N-acetylcysteine, Cyt C: cytochrome c, Cys: cysteine, Glu: glutamic acid.

The present study, through a systematic morphological, molecular, and functional analysis performed in parallel on patients with asymptomatic diverticulosis and complicated DD, for the first time highlighted the possible contribution of mitochondria alterations on the genesis of oxidative stress encountered in DD. Dysfunctional mitochondria in vascular smooth muscle cells (vSMCs) are characterized by the alteration of their morphology, ultrastructure, and membrane permeability and potential [16]. According to this, TEM analyses in the DD specimens revealed the presence of an evident shape alteration, an increase in mitochondrial number, and clear signs of mitochondrial fusion both in longitudinal and circular muscle layers. These mitochondrial alterations, observed independently of DD clinical phenotypes, may be traced back to mitochondrial adaptations in response to disturbing stimuli. In fact, in affected tissues, mitochondrial number increase can potentially be considered a secondary response to the respiratory chain deficiency, beneficial for improving the energy homeostasis lost [17]. Changes in the size and shape of mitochondria are often related to fission (division of a single organelle into two or more independent structures) and fusion (the opposing reaction), events related to the maintenance of mitochondrial integrity and homeostasis [18,19]. Note, in fact, that an imbalance in mitochondrial fission and fusion processes is considered a prerequisite for mitochondrial damage, and an increase in mitochondrial fusion has been observed in hearts failing following ischemia/reperfusion [20]. Based on these results, and in light of recent studies highlighting the dynamic role of mitochondria in smooth muscle cells, particularly in vascular diseases [20], it is therefore reasonable to hypothesize a direct involvement of this organelle in DD pathogenesis whose distinctive signature in all DD clinical phenotypes is mainly represented by oxidative stress damage [5]. Oxidative stress in living organisms reflects an imbalance between the production of radical species and host abilities in their neutralization that leads to damage to cell structures and biological molecules [21]. It has been shown to impact human gastrointestinal smooth muscle in different conditions [22]. In particular, oxidative stress has been shown to induce a phenotypic switch from contractile to synthetic in intestinal vSMCs [5]. This effect has also been observed in vSMCs in cardiovascular and cerebrovascular districts [23,24]. It has been shown that a decline in mitochondrial respiration can lead to ROS overproduction [25,26,27]. Mitochondrial electron transport (ETC) consists of five complexes—I, II, III, IV and V, also called ATP synthase—each of which is constituted by the assembly of more subunits to give the final active structure, and an impairment in this organization could affect ETC functionality leading to ROS overproduction [28]. At least 11 different sites of ROS generation have been identified in isolated mitochondria incubated with different metabolic substrates and inhibitors [29]. The most studied are the ubiquinone binding sites of respiratory complex I and III. As far as complex IV is concerned, its reduced activity has been associated with mitochondrial ultrastructural damage and ROS generation in vSMCs [30]. Although the contribution of complex II to the production of overall mitochondrial ROS is not yet fully understood, recent evidence suggests that complex II-mediated ROS production is relevant under ischemia/reperfusion conditions [31]. Thus, mitochondria are primarily responsible for ROS release, both in physiological and pathological conditions, by ETC activity that transfers electrons derived from the oxidized substrate [26,27]. To assess whether the increase in mitochondrial biogenesis and the ultrastructural and morphological alterations we observed in DD samples could be interpreted as phenomena of adaptation to a stress condition, ETC complexes II and IV were evaluated. In detail, in the present study we evaluated the subunit A of complex II (SDHA), which is encoded by nuclear DNA whose copy number remains stable throughout the life of the cell, and the subunit I of complex IV (COX), which is entirely encoded by mitochondrial DNA and whose copy number is therefore strictly linked to the mitochondrial number and therefore variable [12]. Several insights emerge from these experiments. In the controls, the protein content of both ETC markers, SDHA and COX, was lower in longitudinal smooth muscle cells than in circular smooth muscle cells, in agreement with the lower mitochondria content in the longitudinal layer already reported in previous studies on human tissues [32]. Such quantitative differences between the two muscle layers were not observed in the DD samples, in which an overall decrease in SDHA content was observed in parallel with an increase in COX content. Dysfunction of complex II limits mitochondrial energy production and promotes oxidative stress, contributing to cellular damage, leading to a possible speculation that SDHA decrease could contribute to exacerbating the impaired oxidative status that characterizes DD [33]. In turn, the progressive increase in COX content, whose synthesis is entirely regulated by mitochondrial DNA, could be related to the activation of mitochondrial biogenesis, suggesting the existence of a compensatory mechanism implemented by cells to cope with oxidative imbalance. In fact, the increase in mitochondrial number can help cells to reduce the flow of electrons per unit of mitochondria, reducing ROS production [34]. The increase in mitochondrial mass was ulteriorly confirmed in isolated smooth muscle cells. In this contest, no differences were appreciable between the two muscle layers of the control, possibly related to the different sensitivity of the two methods (flow cytometry and Western blot) and to the different biological samples (muscle tissue and isolated cells) used. In fact, while cytofluorimetric analyses were performed on isolated single smooth muscle cells, Western blots were performed on the entire lysate containing the total protein of all cells present in tissues. However, mitochondria in DD samples presented a collapse of mitochondrial membrane potential independently from DD phenotype, reflecting an altered metabolic status of cells probably related to the altered ETC. The mitochondrial membrane potential is a key indicator of mitochondrial activity, reflecting the processes of electron transport and oxidative phosphorylation driving ATP production. In physiological conditions, its level remains stable, reflecting the normal activity of cells [35]. Several conditions might impair mitochondrial membrane potential; for example, its decrease can occur by ischemia/reperfusion injury insults, a mechanism previously hypothesized to concur in oxidative stress imbalance in DD [5,32]. Secondly, the released ROS can induce the release of ROS, according to the process of “reactive oxygen species (ROS)-induced ROS release” (RIRR), leading to a harmful cycle that results in the collapse of mitochondrial membrane potential [36,37]. In turn, the increase in ROS could lead to the inability of cells to neutralize them, also following the gradual decrease in glutathione content, as observed from diverticulosis to complicated DD, where it becomes more severe depending on the severity of the pathology. Within the non-enzymatic antioxidant category, glutathione represents the most important intracellular defense against ROS [38]. Moreover, among the arsenal of antioxidants present in mitochondria, glutathione represents one of the main lines of defense for the maintenance of the appropriate mitochondrial redox environment [39]. Interestingly, a recent case–control study has brought out the role of gamma-glutamyl transferase, the first enzyme to operate in de novo glutathione synthesis pathway, as a biomarker for colon diverticulosis stressing the relevant role of this antioxidant in predicting cellular oxidative status. Ulteriorly, it was hypothesized that circulant/systemic increase in this enzyme—probably due to the inflammatory process, membrane lesions, or enterocyte apoptosis activation—inversely correlates with its decrease into cellular cytoplasm, as demonstrated in our DD samples [40]. Mitochondrial metabolic changes influence, in turn, cell homeostasis that is directly involved in smooth muscle cell remodeling and, consequently, in their functionality [41,42,43]. The present study relates mitochondrial alterations to the ability of cells to respond to contraction and relaxation stimuli, which is significantly compromised in both DD phenotypes. Finally, the role of oxidative imbalance in DD muscle alterations was substantiated in the present study also by the results obtained from the exposure of DD smooth muscle cells to N-acetylcysteine (NAC). NAC is an antioxidant compound known to have a significant impact on glutathione levels. It effectively increases glutathione content by providing cysteine, a key precursor for its synthesis, thereby reducing oxidative stress [44]. Treatment with this antioxidant restored DD smooth muscle cell relaxation to that was observed in the control samples. The beneficial effects of NAC in reverting human smooth muscle oxidative stress-driven alterations have already been reported in human obese antrum [45] and in LPS-driven colonic smooth muscle alterations [46]. NAC has also recently been reported to contrast with altered glutathione metabolism affecting gut matrix metalloproteinase in Crohn’s myofibroblasts [47], which are enzymes that have been reported to be also involved in DD pathogenesis [48]. Recent preclinical studies have explored the effects of NAC on mitochondria, focusing on its potential to protect mitochondrial function, reduce oxidative stress, and influence cellular health with particular focus on mitochondrial protection and function, biogenesis, and neuroprotection [49]. The effect of NAC at the mitochondrial level is schematically reported in Figure 5.

In vivo, a recent pilot study showed that oral NAC administration in human older adults could prevent oxidative stress and mitochondrial dysfunction, promoting a generally healthy status [44]. Similarly, some studies have reported clinical use of NAC in respiratory diseases, paracetamol poisoning, central nervous system disorders, and cardiovascular diseases, in which the positive effects were associated with an increase in glutathione concentration, as observed in preclinical studies [50]. Interestingly, and in agreement with the results reported here, it was observed that NAC was able to play a protective role in ischemia/reperfusion conditions by preserving mitochondrial function by maintaining the correct assembly and functional properties of the mitochondrial respiratory chain while reducing ROS generation, consequently restoring energy efficiency [51]. The main limitation of the present study could be represented by the inextensive evaluation of NAC’s effects on the revision of mitochondrial parameters. Such an approach could suggest the presence of irreversible damage at the mitochondrial level and/or indicate the need to use mitochondrial-specific antioxidants or a cocktail of antioxidants with different mechanisms of action. However, considering the reversal of oxidative imbalance and the restoration of cell functional activity by NAC, antioxidant therapy remains a potential therapeutic strategy and research topic to invest in.

## 4. Materials and Methods

### 4.1. Patients and Colonic Specimens

Sigmoid colonic specimens were obtained from patients undergoing sigmoid resection for complicated diverticular disease (cDD group), non-obstructing colon cancer with the occasional finding of diverticula (asymptomatic diverticulosis: DIV group), and non-obstructing colon cancer without macroscopic diverticula (control group). In detail, the controls were taken from the macroscopically normal area not affected by cancer and approximately 5–6 cm away from the neoplastic area. All patients underwent surgery at the Sapienza University Hospital Policlinico Umberto I of Rome. This study was approved by the formal Ethical Committee, CET—Lazio Territorial Ethics Committee Area 1 (Code: CE4702-2017) and conducted according to the ethical guidelines of the 1975 Declaration of Helsinki (6th revision, 2008). All patients gave informed consent.

### 4.2. Cellular Ex Vivo Mode

Under sterile conditions, the mucosa/submucosa and the longitudinal and circular layers were separated, and smooth muscle cells (SMC) were isolated as previously described by Tattoli [52]. Briefly, 500 µm slices of longitudinal and circular muscle layers cut by Thomas Scientific blades (FisherScientific) were incubated overnight in DMEM F-12 supplemented with penicillin–streptomycin solution (10,000 U/mL), gentamicin (1 mg/mL), amphotericin B (250 μg/mL), ATP-regenerating system (ATP 3 mM, phosphocreatine 10 mM, creatine phosphokinase 10 U/mL), CaCl_2_ (1 mM), and collagenase (150 U/mL). On the following day, the digested muscle was suspended in DMEM F-12 supplemented with antibiotics for 20 min to allow spontaneous dissociation of SMC. The cells were then harvested and used either immediately or maintained in suspension for up to 2 h for the in vitro model.

### 4.3. Transmission Electron Microscopy (TEM)

The tissues were fixed with 2.5% glutaraldehyde in 0.1 M sodium cacodylate buffer (pH 7.2) for 1 h at room temperature. After washings, the tissues were post-fixed in 1% osmium tetroxide (OsO_4_) in 0.1 M sodium cacodylate buffer for 1 h at room temperature. The samples were then washed in the same buffer, dehydrated through serial ethanol solutions (30–100%), and embedded in an Agar Resin Kit (Agar Scientific, R1031). Using a UC6 ultramicrotome (Leica), ultrathin sections were made, subsequently stained with uranyl acetate and Reybolds lead citrate and examined at 100 kV with a Philips EM 208S transmission electron microscope (Thermo Fisher Scientific, Sugar Land, TX, USA) equipped with a Megaview III SIS camera system (Olympus-SIS Milan, Italy) and iTEM3 software version 3.0.

### 4.4. Western Blot

Smooth muscle tissues were lysed in RIPA buffer (Thermo Scientific™) and Halt™ Protease Inhibitor Cocktail 1X and allowed to stand for 20 min at 4 °C. The tissue suspension was disrupted by a manual homogenizer and then centrifuged for 5 min at 1300× *g* to remove nuclei and large cellular debris. A standard curve was prepared with Albumin standard (Thermo Fisher Scientific™ Sugar Land, TX, USA; 23209), and the total protein extract was evaluated with the Bradford dye reagent assay (Bio-Rad, 500-0006). A total of 30 μg/lane of clarified protein lysate was subjected to Bolt™ Bis-Tris Plus Mini Protein Gels, 4–12% (Invitrogen™, NW04120BOX). Bolt™ MES SDS Running Buffer 20X (Invitrogen™, Thermo Fisher Scientific™ Sugar Land, TX, USA; B0002) was used as the running buffer. Proteins were electrophoretically transferred onto polyvinylidene difluoride (PVDF) membranes (Invitrogen™, LC2002); blocked with 5% defatted dried milk in PBS containing 0.05% Tween-20; and probed with MitoBiogenesis™ Western Blot Cocktail (Abcam, Cambridge, UK; ab123545) containing MAb subunit I of Complex IV (COX), MAb subunit A of succinate dehydrogenase (SDH), and MAb beta-actin. Primary bound antibodies were probed with anti-mouse IgG (Invitrogen™, 31430) and visualized with SuperSignal™ West Pico PLUS Chemiluminescent Substrate (Thermo Scientific, 34579). SeeBlue Plus2 prestained protein standard (LC5925, Invitrogen) was loaded on the gel as a marker. Images were acquired with ChemiDoc Imaging System (BioRad, 94547 Hercules, CA, USA).

### 4.5. Flow Cytometry Analysis

Aliquots of primary SMC suspension culture were centrifuged at 900× *g* 10 min at room temperature, and the pellet was incubated for 10 min at 37 °C in the specific staining solution constituting a dilution of the specific probe in prewarmed PBS. In detail, mitochondrial mass was evaluated by staining cells with MitoTracker™ Green FM (Invitrogen™, M7514) used at a final concentration of 20 nM. Mitochondrial membrane potential was evaluated with Tetramethylrhodamine-Methyl Ester Perchlorate (TMRM) (Invitrogen™, T668) used at a final concentration of 100 nM. Intracellular glutathione (GSH) was detected with CellTracker™ Green CMFDA Dye (Invitrogen™, C2925) used at a final concentration of 0.5 µM. After incubation, the samples were immediately analyzed by FACScalibur cytometer (BD Biosciences Inc., San Diego, CA, USA) equipped with a 488 nm Argon laser and with a 635 nm red diode laser and analyzed using CellQuest Pro software version 6.0 (BD Biosciences, 20161 Milano, Italy). The median values fluorescence intensity histograms of 10,000 events acquired were used to provide a semi-quantitative assessment.

### 4.6. Functional Study

Contraction and relaxation were measured on SMC, as described previously [40]. For contraction studies, 0.5 mL of SMC was added to 0.2 mL of incubation medium containing a maximal dose of Ach (1 µM), and the reaction terminated after 30 s, the time required for the peak contraction, with 1% acrolein. The contraction was expressed as a percentage decrease in cell length from the control taken as 100. For relaxation studies, 0.5 mL of SMC was added to 0.2 mL of incubation medium containing a maximal dose of VIP (1 µM). VIP was added 60 s before Ach, and the reaction was terminated after a further 30 s by the addition of 1% acrolein. Relaxation was measured in SMC pre-exposed to VIP and then contracted with a maximal contraction. In the measurement of the control cell length, the agent was omitted, and an equivalent volume of medium was added. The length of 50 cells in sequential microscopic fields was measured by image-scanning micrometry both in the control state and upon addition of tested agents, using a High Grade Moticam^®^ S6 camera with Motic Images Plus 3.0 application software (Motic Asia, Kowloon Bay, Kowloon, Hong Kong) installed on a phase-contrast microscope (Leica Microsystems, Wetzlar, Germany).

### 4.7. Antioxidant In Vitro Model

Immediately after dissociation, the pure suspension cell culture was treated with antioxidant N-acetylcysteine 25 µM for 2 h at 37 °C/5% CO_2_ [46,53]. To check the potential beneficial effect of antioxidants on the treated cells, a functional study and flow cytometry analyses were performed.

### 4.8. Statistical Analyses

Clinical and demographic data were expressed as median (95%CI). Experimental data were expressed as mean ± SEM of *n* experiments, “*n*” referring to the number of individual patients from whom the colonic specimens were obtained. The different pathological groups of patients with no Gaussian distribution were analyzed using the Kruskal–Wallis test, followed by multiple pairwise comparisons adjusted with Dunnett’s corrections. The significance level was set at *p* < 0.05 (*). Comparison between the longitudinal and circular muscles of the same patients and comparison between the treated and untreated samples for the in vitro model were performed by paired *T*-tests. The significance level was set at *p* < 0.05 (*). Numerical estimates were obtained with GraphPad Prism version 5.00 for Windows, GraphPad Software 5, San Diego, CA, USA.

## 5. Conclusions

In conclusion, the present study highlights that the predominant transmural oxidative imbalance that characterizes the DD specimens is associated with mitochondrial alterations. These, already present in diverticulosis, consist of mitochondrial chain damage (loss of ECT complex II and mitochondrial membrane potential) associated with compensatory mitochondrial biogenesis (increase in ECT complex IV and mitochondrial mass). It is possible to speculate that in the early stages of DD pathogenesis, these mitochondrial alterations act as an adaptive response to anaerobic stress induced by ischemia/reperfusion damage to optimize oxygen deficiency, similar to what occurs in cardiac hypertrophy [54]. Progressively, in complicated DD, this adaptive response leads to mitochondrial oxidative imbalance and excessive consumption of antioxidant reserves (depletion of intracellular glutathione), with consequent functional muscle damage. Of utmost importance is that muscle impairment is restored, in the ex vivo model, by a NAC-induced increase in glutathione levels. It is therefore clear that oxidative stress has a key role in the development and exacerbation of DD through the promotion of tissue damage in the colon. Tailored therapeutic strategies targeting oxidative stress, including antioxidant therapy, may be considered as potential ways for DD management still not tested for this disease.

## Figures and Tables

**Table 1:** Human tissue collection.

	Controls	Diverticulosis	Complicated DD
**Gender**	7 women/6 men	8 women/9 men	8 women/7 men
**Age (years) median (95% CI)**	68 (53–92)	76 (46–89)	67 (42–85)
**BMI (kg/m^2^) median (95% CI)**	23 (21–30)	24.0 (21–29)	26.0 (20–34)

**Table 2:** Cellular functional activity in smooth muscle cells evaluated by micrometric assay.

	*Contraction*	*Relaxation*
***LONGITUDINAL layer***		
**DIV**	45.7% ± 7.1	56.9% * ± 5.3
**cDD**	61.7% * ± 9.6	56.9% * ± 5.3
***CIRCULAR layer***		
**DIV**	46.7% ± 5.8	21.9% ± 10.2
**cDD**	56.4% ± 14.2	54.6% ± 13.6

Data are expressed as mean ± SEM of percentage decrease vs. controls (*n* = 4–7). * *p* = 0.001 vs. CTR. CTR: control, DIV: diverticulosis, cDD: complicated diverticular disease.

## Data Availability

The data presented in this study are available on request from the corresponding author.

## Supplementary Materials

The following supporting information can be downloaded at https://www.mdpi.com/article/10.3390/ijms25179680/s1.
