# Supplementary material for: The Unexplored Role of Mitochondria-Related Oxidative Stress in Diverticular Disease
Source: Int J Mol Sci. 2024 Sep 6;25(17):9680. doi: 10.3390/ijms25179680 (PMC11395029; doi:10.3390/ijms25179680)
Supplement: Supplementary file 1 [file ijms-25-09680-s001.zip › Supplementary Figure S1.pdf]

Original Blot of Western Blotting Assay

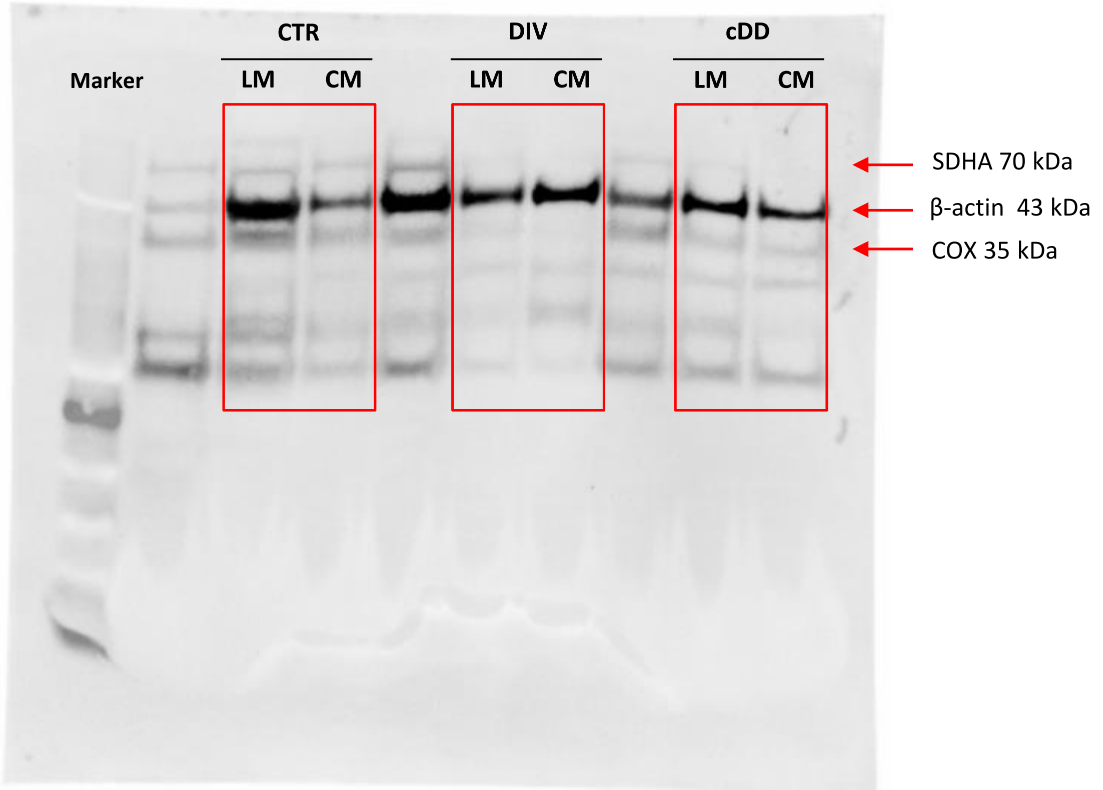

Supplementary Figure S1: Original Blot of SDHA, β-actin and COX proteins
