# Supplementary material for: The Unexplored Role of Mitochondria-Related Oxidative Stress in Diverticular Disease
Source: Int J Mol Sci. 2024 Sep 6;25(17):9680. doi: 10.3390/ijms25179680 (PMC11395029; doi:10.3390/ijms25179680)
Supplement: Supplementary file 1 [file ijms-25-09680-s001.zip › Supplementary Table S1.pdf]

Supplementary Table 1. Cellular functional activity in smooth muscle cells evaluated by micrometric assay

LENGTH (μM)	CTR	DIV	cDD
LONGITUDINAL	61.96±4.03	63.37±3.93	61.82±5.12
CIRCULAR	61.02±4.05	62.70±4.72	61.28±4.37

Data are expressed as mean±SE of percentage decrease of controls, 4-7 experiments. CTR (control), DIV (diverticulosis), cDD (complicated diverticular disease).
